# 666. Does Platelet-rich Plasma-enhanced Fat Grafting Improve Outcomes in Post-burn Scarring? A Retrospective Comparative Study

**DOI:** 10.1093/jbcr/irag033.532

**Published:** 2026-04-06

**Authors:** S Kumar Sailesh, I, Tapasya Katta, Elizabeth Hale, Catalina Breton, Sherry S Collawn

**Affiliations:** University of Alabama at Birmingham, Birmingham, AL; University of Alabama at Birmingham, Birmingham, AL; University of Alabama at Birmingham, Birmingham, AL; University of Alabama at Birmingham, Birmingham, AL; University of Alabama at Birmingham, Birmingham, AL

## Abstract

**Introduction:**

Fat grafting has emerged as a powerful tool for burn reconstruction, yet outcomes remain unpredictable with variable graft retention. Platelet-rich plasma (PRP), containing growth factors that promote angiogenesis, may enhance graft survival in reconstructive surgery. The role of PRP in burn patients undergoing fat grafting remains poorly defined. This study compares clinical outcomes and patient satisfaction between burn patients treated with fat grafting with versus without PRP.

**Methods:**

A single-centre retrospective cohort was performed on burn patients who underwent autologous fat grafting with or without PRP augmentation (Jan 2019 and July 2024). Indications included post-burn hypertrophic scars, keloids, and scar contractures. PRP was mixed with fat at a ratio of 0.2:0.8 mL. Patient demographics, complications, and patient satisfaction scores (5-point Likert scale) were recorded. Statistical analysis was performed with significance at *p*<.05.

**Results:**

Seventy-three burn patients were analyzed (PRP: n = 51; non-PRP: n = 22) with median follow-up 13 months. Groups were comparable in age (mean 42.1 vs. 37.7 years, *p*=.11). The PRP group demonstrated significantly higher baseline risk factors: elevated BMI (mean 32.9 vs. 26.5, *p*=.04), increased hypertension prevalence (43% vs. 14%, *p*=.03), and substance use (47% vs. 23%, *p*=.05).

Despite higher-risk, complication profiles were similar between groups. No infections, seromas, or hematomas occurred in either cohort. Rates of ecchymosis (12% vs. 9%, *p*=.7), numbness (6% vs.14%, *p*=.2), dermatitis (10% vs.5%, *p*=.4), fat necrosis (2% vs.0%, *p*=.5), and wound dehiscence (6% vs.18%, *p*=.1) showed no significant differences. The need for repeat fat grafting procedures was comparable (37% vs. 45%, *p*=.5).

Patient satisfaction scores at final follow-up were significantly higher in the PRP group (3.57 vs. 3.26, *p*=.03), despite similar early satisfaction at 1 month (3.91 vs.3.9, *p*=.71), suggesting sustained benefit over time.

**Conclusions:**

In burn reconstruction, PRP-augmented fat grafting demonstrated superior long-term patient satisfaction despite patients having significantly higher baseline risk factors including elevated BMI, hypertension, and alcohol use. Complication rates were equivalent between groups, suggesting PRP may mitigate adverse effects of high-risk comorbidities in challenging burn reconstruction cases.

**Applicability of Research to Practice:**

For surgeons managing high risk patients with obesity, hypertension or substance use, PRP offers a low-risk intervention that may counteract these adverse factors. The sustained improvement in patient satisfaction at final follow-up, not evident early postoperatively, suggests PRP's benefits emerge over time through enhanced tissue integration. This time-dependent effect warrants investigation of PRP's impact on long-term scar remodeling, outcomes that matter most to burn survivors.

**Funding for the study:**

N/A.

